# Effect of Transcutaneous Auricular Vagus Nerve Stimulation on Protracted Alcohol Withdrawal Symptoms in Male Alcohol-Dependent Patients

**DOI:** 10.3389/fpsyt.2021.678594

**Published:** 2021-08-30

**Authors:** Yong-Mei Wang, Ya-Yun Xu, Yi Zhai, Qian-Qian Wu, Wen Huang, Yan Liang, Yan-Hong Sun, Lian-Yin Xu

**Affiliations:** ^1^Department of Nursing, Affiliated Psychological Hospital of Anhui Medical University, Hefei, China; ^2^Anhui Mental Health Center, Hefei, China; ^3^Department of Epidemiology and Biostatistics, School of Public Health, Anhui Medical University, Hefei, China; ^4^Department of Material Dependence, Hefei Fourth People's Hospital, Hefei, China; ^5^Hefei Medical Research Centre on Alcohol Addiction, Hefei, China; ^6^Department of Pharmacy, Hefei Fourth People's Hospital, Hefei, China

**Keywords:** alcohol dependence, brain-derived neurotrophic factor, depression, protracted alcohol withdrawal symptoms, sleep quality, transcutaneous auricular vagus nerve stimulation

## Abstract

Protracted alcohol withdrawal symptoms (PAWS), characterized by the presence of substance-specific signs and symptoms (including anxiety, irritability, mood instability, insomnia, and cravings), make alcohol abstinence difficult and increase the risk of relapse in recovering alcoholics. The goal of this study was to evaluate the effect of transcutaneous auricular vagus nerve stimulation (taVNS) on PAWS and plasma brain-derived neurotrophic factor (BDNF), interleukin-6 (IL-6), tumor necrosis factor-α (TNF-α), and leptin levels in patients with alcohol dependency. A total of 114 patients with alcohol dependence were randomly divided into two groups: the treatment group and the control group. The patients in the treatment group were treated with taVNS of the bilateral auricular concha using an ear vagus nerve stimulator. The Pennsylvania Alcohol Craving Scale was used to evaluate the extent of craving for alcohol. The Self-Rating Anxiety Scale and Self-Rating Depression Scale (SDS) were used to evaluate the extent of anxiety and depression symptoms, respectively. The Pittsburgh Sleep Quality Index (PSQI) was used to assess sleep quality. Enzyme-linked immunosorbent assay was used to measure plasma BDNF, IL-6, TNF-α, and leptin levels. The results showed that the SDS and PSQI scores were significantly lower in the treatment group than in the control group. Moreover, compared with the control group, the average BDNF levels in the treatment group were significantly increased. These results suggest that taVNS could improve the depression symptoms and sleep quality in alcohol-dependent patients after withdrawal, which might be related to the upregulation of plasma BDNF levels.

## Introduction

It has been estimated that 3 million people die every year due to the harmful use of alcohol according to the data of the World Health Organization, which causes great health and economic losses to individuals and society. A high relapse rate is a difficult problem in the treatment of patients with alcohol dependence. It has been reported that ~90% of alcoholics may experience at least one relapse within 4 years after treatment (1), which may be related to protracted alcohol withdrawal symptoms (PAWS) (2).

PAWS, a cluster of symptoms that occurs because of the sudden cessation of alcohol consumption after chronic or prolonged ingestion, includes anxiety, irritability, mood instability, insomnia, and craving (3). These withdrawal symptoms make attempts to abstain from alcohol difficult and increase the risk of relapse in recovering alcoholics. Therefore, it is of great significance to prevent relapse by reducing withdrawal symptoms in patients with alcohol dependence. At present, drug therapy is mainly used to treat PAWS in patients with alcohol dependency. Given that the liver and kidney functions of alcohol-dependent patients are impaired, the use of drugs may further increase the burden of the liver and kidney. Thus, there is an urgent need to find a safe method to treat PAWS.

Non-invasive transcutaneous auricular vagus nerve stimulation (taVNS) stimulates the auricular branch of the vagus nerve through the outer ear in humans by placing an electrode on the corresponding auriculate acupoint (4). This method avoids trauma pain and fear of acupuncture, making it more easily accepted by patients (5). There is evidence that intermittent and chronic stimulation of taVNS is widely used to treat drug-resistant epilepsy (6), depression (7, 8), and insomnia (9). Because of its good safety and potential therapeutic effects on withdrawal symptoms, taVNS may be a useful, non-pharmacological, and non-invasive approach that yields beneficial effects for PAWS management in individuals with alcohol dependence.

Brain-derived neurotrophic factor (BDNF) is associated with many neuropsychiatric diseases, including alcohol use disorders and depression. It has been shown that the level of BDNF in patients with alcoholism was significantly lower than that in healthy volunteers (10). A further study indicated that the level of BDNF in the serum of alcohol-dependent patients after abstinence was significantly lower than that before abstinence (11). Moreover, low serum BDNF has been found to be associated with suicidal ideation in major depressive disorder (12). A large number of studies have shown that vagus nerve stimulation (VNS) improves the symptoms of several diseases including ischemic stroke (13), Parkinson's disease (14), and depression (15), possibly in conjunction with the upregulated expression of BDNF. Thus, the effect of taVNS on plasma BDNF levels and the relationship between PAWS and plasma BDNF levels were investigated in the present study.

Elevated peripheral immune factors have been demonstrated to be associated with altered mood and memory in patients with current or recent alcohol dependence (16). Moreover, the levels of interleukin-6 (IL-6) and tumor necrosis factor-α (TNF-α) in peripheral blood have been observed to be positively correlated with craving during alcohol withdrawal (17). Considering that recent data have suggested the anti-inflammatory role of VNS (18), whether these inflammatory cytokines could be affected by taVNS in alcohol-dependent patients was also evaluated in the present study.

Leptin, the first discovered and the most studied adipokine, maintains energy homeostasis and controls body weight (19). Accumulating evidence has shown that leptin may modulate withdrawal-induced craving in alcoholic individuals (20, 21). Serum leptin levels have been reported to be altered in alcohol-dependent patients (22, 23). Moreover, a close relationship between serum leptin levels and alcohol craving in both sexes has been observed (24). However, no studies have been conducted on the relationship between leptin and PAWS in patients with alcohol dependency. In addition, the serum levels of leptin have been observed to be decreased in animals that received VNS compared to control animals (25). Thus, the effect of taVNS on plasma leptin levels in patients with alcohol dependency was investigated.

Based on these findings, we hypothesized that taVNS could ameliorate PAWS in alcohol-dependent patients, the mechanism of which may be related to its regulation of plasma BDNF, IL-6, TNF-α, and leptin levels. To test this hypothesis, a total of 114 patients with alcohol dependence were randomly divided into two groups: the treatment and control groups. The patients in the treatment group were treated with taVNS of the bilateral auricular concha using an ear vagus nerve stimulator. The Pennsylvania Alcohol Craving Scale (PACS), Self-Rating Anxiety Scale (SAS), Self-Rating Depression Scale (SDS), and Pittsburgh Sleep Quality Index (PSQI) were used to evaluate the extent of PAWS. Enzyme-linked immunosorbent assay (ELISA) was used to measure plasma BDNF, IL-6, TNF-α, and leptin levels.

## Materials and Methods

### Patients

This study was conducted at the Hefei Fourth People's Hospital, Anhui Mental Health Center, between September 2019 and December 2020. A total of 114 patients with alcohol dependence were enrolled by an experienced and trained researcher (Yi Zhai) in accordance with the guidelines of the structured clinical interview according to the “International Classification of Diseases 10th Revision” criteria. All the patients were male inpatients. All of them were in the drinking period when they were sent to the hospital. They underwent the benzodiazepine substitution for decreasing dependence therapy (for ~1 week) and were in a stable period of alcohol withdrawal at the time of enrollment. The criteria for joining the group were as follows: (1) age 18 to 65 years, (2) currently not receiving any medication, and (3) gave written informed consent to participate in the study. The exclusion criteria were as follows: (1) serious heart, liver, or kidney diseases, and (2) experiencing other mental illnesses. In accordance with the principles of the Declaration of Helsinki, all patients provided informed written consent prior to participation. This study was approved by the Ethics Committee of the Hefei Fourth People's Hospital, Anhui Mental Health Center.

### Study Design

A total of 114 patients with alcohol dependence were randomly divided into two groups: the treatment group (*n* = 58) and control group (*n* = 56). The patients in the treatment group were treated with taVNS of the bilateral auricular concha using an ear vagus nerve stimulator. The patients in the control group wore the same instrument without power. All the patients underwent the benzodiazepine substitution for decreasing dependence therapy (for ~1 week) and were in a stable period of alcohol withdrawal at the time of enrollment before taVNS treatment or negative stimulation. The taVNS device was operated according to a previously described method (26). Briefly, the stimulation frequency of the taVNS was 20 Hz, and the intensity was increased gradually from 0.4 to 1.0 mA according to the tolerance of the patient. Transcutaneous stimulation was performed three times a day, 30 min per session. The treatment period was 4 weeks. The blood samples and scale scores of all the patients were collected before and after the treatment.

### Clinical Data and Scale Score Measurements

The PACS was used to evaluate the extent of the craving for alcohol. The SAS and SDS were used to evaluate the extent of anxiety and depression symptoms, respectively. The PSQI was used to assess sleep quality. A demographic questionnaire was used to collect general information about the participants. According to the conclusion of the other studies (27, 28), the boundary values of the SAS scale anxiety state and the SDS scale depression state were both 50 points. In terms of PSQI, scores > 5 indicate poor sleep quality (29).

### Biochemical Measurements

Blood samples were taken from the vein of the participant between 8:00 a.m. and 9:00 a.m. at the end of an overnight fasting period of 10–11 h at baseline before taVNS treatment and on the next morning after last taVNS treatment. Tubes containing ethylenediaminetetraacetic acid were used to collect the samples. The blood samples were immediately centrifuged at 3,000 rpm for 5 min at 4°C. The supernatant was extracted as the plasma sample. The extracted plasma was stored at −80°C until detection. Commercially available ELISA kits were used to measure the plasma concentrations of leptin, BDNF, IL-6, and TNF-α (Jianglai Bio, Shanghai, China) according to the instructions of the manufacturer. The assay sensitivities for leptin, IL-6, TNF-α, and BDNF were 0.1, 10, 1.0, and 1.0 pg/ml, respectively, with an intra-assay variation of <9.0% and an inter-assay variation of <11.0%.

### Sample Size

The sample size was calculated according to the changes in the depression scores based on the study conducted by Tseng et al. (30). It was calculated considering a 95% confidence interval, 80% power (α = 0.05, β = 0.2), and the related mean and standard deviation (SD) of the depression scores in the aforementioned study (μ1 = 5.35; μ2 = 8.58; SD1 = 2.08; SD2 = 3.53). The minimum sample size was 51 for each group. A total of 114 patients were recruited for the present study.

### Statistical Analysis

The data were analyzed using SPSS (version 17.0; IBM Corp., Armonk, NY, USA). The differences in the baseline measurements between the two groups were analyzed using a *t*-test. After a 4-week treatment, an analysis of covariance (ANCOVA) was performed to compare the plasma concentrations of leptin, BDNF, IL-6, and TNF-α and the scores of the scales between the two groups, controlling for the respective baseline measurements by using these variables as covariates. The statistical significance was set at *p* < 0.05.

## Results

### Baseline Data of Demographic Values; Alcohol-Related Data; Plasma BDNF, IL-6, TNF-α, and Leptin Levels; and Scales of the Treatment and Control Groups

As shown in Table 1, there were no significant differences in the age, body mass index, educational status, years of drinking, or the daily intake between the two groups.

**Table 1:** Comparison of baseline data of mean values (or ratios) of demographic data, alcohol-related data, scale scores, and plasma BDNF, IL-6, TNF-α, and leptin levels in the treatment and control groups (mean ± SEM).

**Variables**	**Control group**	**Treatment group**	**Statistics (t/χ^2^)**	***P***
Age	41.38 ± 0.87	40.00 ± 0.89	1.094	0.277
BMI (kg/m^2^)	21.55 ± 0.41	22.02 ± 0.36	0.867	0.387
Educational status (years)	10.02 ± 0.48	10.47 ± 0.49	0.658	0.512
Years of drinking	17.35 ± 0.94	15.78 ± 0.98	1.157	0.250
Daily intake (standard drink)	7.00 ± 1.26	6.41 ± 0.47	0.546	0.588
PACS score	7.25 ± 0.59	7.96 ± 0.83	0.701	0.485
SDS score	46.87 ± 1.36	49.03 ± 1.62	0.111	0.307
SAS score	43.33 ± 1.15	45.43 ± 1.33	1.198	0.233
PSQI score	6.86 ± 0.54	7.67 ± 0.59	0.348	0.316
leptin (ng/ml)	8.47 ± 0.24	8.12 ± 0.21	1.114	0.267
IL-6 (pg/ml)	30.22 ± 0.70	29.71 ± 0.55	0.588	0.558
TNF-α (pg/ml)	49.72 ± 1.23	49.03 ± 1.14	0.408	0.684
BDNF (ng/ml)	41.95 ± 0.74	40.36 ± 0.72	1.517	0.132

*BMI, body mass index; SEM, standard error of mean*.

In terms of scales, compared with the control group, there was no difference in the average scores of the PACS, SDS, SAS, and PSQI in the treatment group (Table 1). With 50 points as the critical value (both for SAS and SDS), 18 people were considered to have anxiety symptoms, with a prevalence of 15.8% (18/114), and 20 people were considered to have depression symptoms, with a prevalence of 17.5% (20/114). With 5 points as the critical value for PSQI, 59 people were considered to have sleeping problems, with a prevalence of 51.8% (59/114).

Table 1 shows that the differences between the groups were not statistically significant in terms of plasma leptin, IL-6, TNF-α, and BDNF levels.

### Effects of Auricular Acupressure on the Scores of Depression-, Anxiety-, Craving-, and Sleep Quality-Related Scales

**Figure 1:**
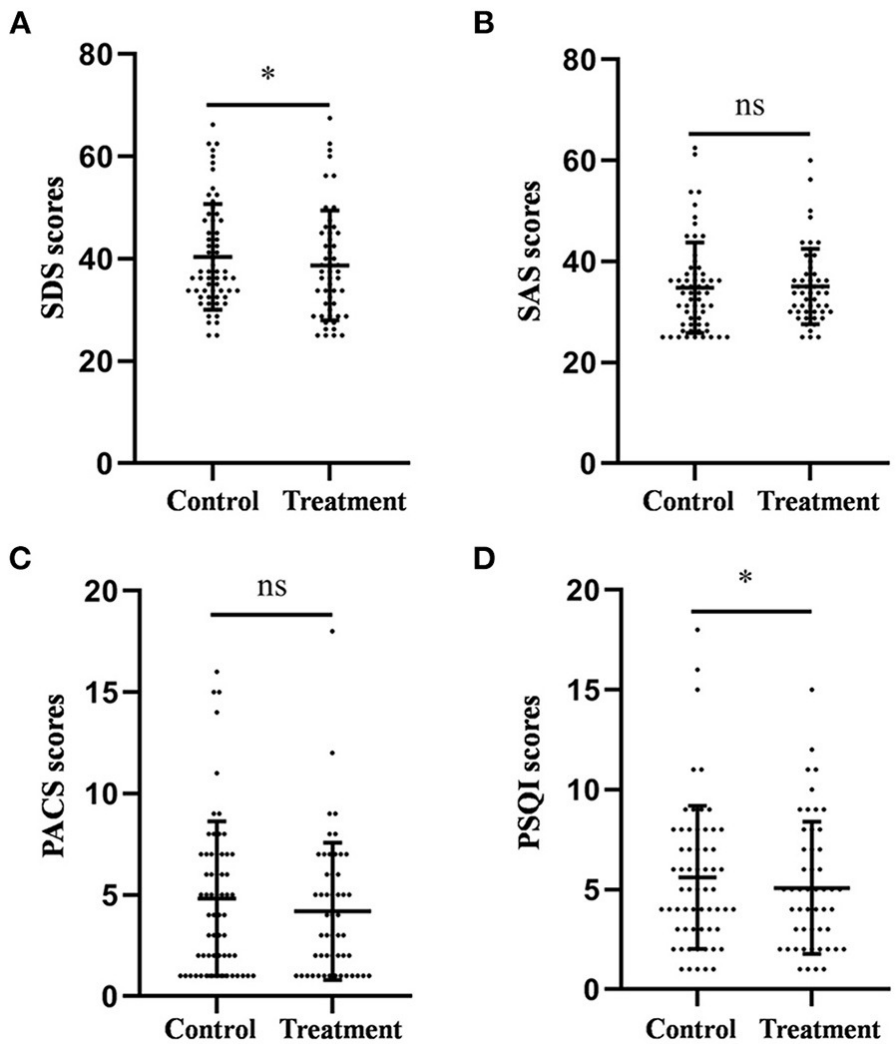
Comparison of the scores of the SDS **(A)**, SAS **(B)**, PACS **(C)**, and PSQI **(D)** between the treatment group and the control group after 4-week treatment. *P < 0.05, was considered statistically significant.

The average SDS scores of the control and treatment groups were 40.38 ± 1.31 and 37.11 ± 1.29, respectively. As shown in Figure 1, the results of the ANCOVA showed that the SDS score in the treatment group was significantly lower than that in the control group (*F* = 5.987, *p* = 0.016, Figure 1A).

The average PSQI scores of the control and treatment groups were 5.61 ± 0.46 and 4.88 ± 0.43, respectively. Similarly, the PSQI score in the treatment group was significantly lower than that in the control group (*F* = 4.104, *p* = 0.045, Figure 1D).

The average SAS scores of the control and treatment groups were 34.80 ± 1.15 and 35.05 ± 1.05, respectively. The average PACS scores of the control and treatment groups were 4.82 ± 0.49 and 3.92 ± 0.39, respectively. The results of the ANCOVA showed that there was no difference in the scores of anxiety (*F* = 0.314, *p* = 0.576, Figure 1B) and craving (*F* = 1.567, *p* = 0.214, Figure 1C) between the two groups.

Taken together, these results suggest that auricular acupressure could improve depression and sleep quality; however, it had no significant effect on anxiety and craving in patients with alcohol dependence after withdrawal.

### Effects of Auricular Acupressure on the Plasma BDNF, IL-6, TNF-α, and Leptin Levels

**Figure 2:**
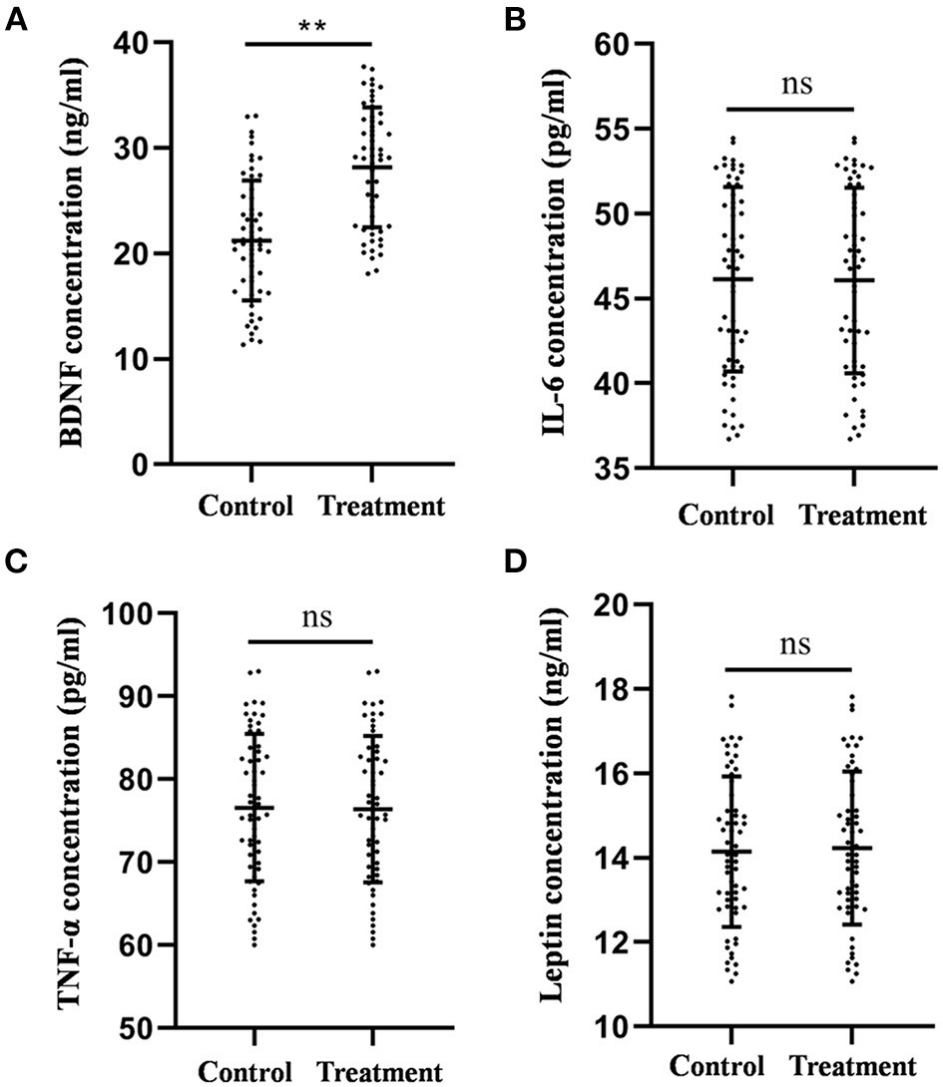
Comparison of the plasma BDNF **(A)**, IL-6 **(B)**, TNF-α **(C)**, and leptin **(D)** levels between the treatment group and the control group after 4-week treatment. **P < 0.01, was considered statistically significant.

The mean BDNF levels in the control and treatment groups were 21.23 ± 0.73 and 28.17 ± 0.76 ng/ml, respectively. As shown in Figure 2, the results of the ANCOVA showed that the mean BDNF levels were significantly higher in the treatment group than in the control group (*F* = 38.978, *p* < 0.001, Figure 2A).

The mean IL-6 levels in the control and treatment groups were 46.13 ± 0.71 and 46.06 ± 0.73 pg/ml, respectively. The mean TNF-α levels of the control group and the treatment group were 76.58 ± 1.16 and 76.40 ± 1.18 pg/ml, respectively. The mean leptin levels in the control and treatment groups were 14.15 ± 0.23 and 14.23 ± 0.24 ng/ml, respectively. No statistical difference was observed in IL-6 (*F* = 0.058, *p* = 0.210, Figure 2B), TNF-α (*F* = 0.030, *p* = 0.862, Figure 2C), and leptin (*F* = 0.039, *p* = 0.843, Figure 2D) levels between the two groups according to the results of the ANCOVA.

Collectively, these results indicate that auricular acupressure could increase plasma BDNF levels; however, it had no significant effect on the levels of IL-6, TNF-α, and leptin.

## Discussion

The present study is the first to demonstrate that taVNS could improve depression and sleep quality in patients with alcohol dependence after withdrawal. Moreover, the results of the ANCOVA showed that the plasma BDNF levels were significantly increased in patients receiving taVNS treatment. These results suggest that taVNS could improve depression symptoms and sleep quality in alcohol-dependent patients after withdrawal, which might be related to the upregulation of plasma BDNF levels.

PAWS, a response to the reduction or termination of alcohol exposure, is considered the core feature of patients with alcohol dependence (31). Negative affective states, such as anxiety and depression, and decline in the cognitive function associated with alcohol withdrawal represent the cause of excessive alcohol consumption and may explain a relapse into alcoholism (32). The PACS, one of the most widely used tools to measure the craving for alcohol, has demonstrated reliability and validity in numerous studies (33, 34). The SAS and SDS are widely used as screening tools, covering affective, psychological, and somatic symptoms associated with anxiety and depression, respectively (21, 35). The PSQI, a 19-item self-report questionnaire that measures subjective sleep quality, has shown good reliability and validity for both healthy and clinical groups with mental and physical health problems (36). Therefore, to evaluate the degree of PAWS in patients with alcohol dependence after withdrawal, the PACS, SAS, SDS, and PSQI were selected to assess the extent of craving for alcohol, anxiety symptoms, depression symptoms, and sleep quality, respectively.

taVNS, as a non-invasive intervention, has beneficial effects on a wide range of neurological, psychiatric, and medical conditions based on clinical observations. Animal experiments and clinical trials have demonstrated that taVNS in the auricular concha region elicits therapeutic effects on depression symptoms (37, 38). Moreover, VNS was approved by the United States Food and Drug Administration in 2005 for treating chronic refractory depression (39). Furthermore, the 2014 “Evidence-based Guidelines of Clinical Practice with Acupuncture and Moxibustion: Depression” also recommended electroacupuncture treatment and auricular acupuncture as treatments for depression (40). Consistently, in the present study, the patients who received taVNS treatment showed lower SDS scores compared with the control patients, indicating that taVNS effectively improved the depression symptoms of alcohol-dependent patients after withdrawal.

taVNS has shown promising effectiveness in treating primary insomnia (41). Another clinical study confirmed that taVNS can alleviate insomnia in patients with depression (42). In line with these results, the PSQI scores were significantly lower in the treatment group than in the control group, providing more data for taVNS to improve sleep quality in alcohol-dependent patients.

Studies on the effects of taVNS on anxiety are limited and controversial. taVNS has been shown to be effective in treating anxiety symptoms in depressed patients (42). However, taVNS has been reported to not affect the generalization and physiological indices of fear, a typical hallmark of anxiety disorders (43). The results of the present study first confirmed that taVNS had no significant effect on anxiety symptoms in patients with alcohol dependence. Similarly, there was no difference in the craving scores between the two groups. Numerous studies have demonstrated that anxiety is widely considered to be causally related to alcohol craving and consumption, as well as the development and maintenance of alcohol use disorder (44). Thus, it is rational to presume that the lack of effect of taVNS on craving may be related to the fact that taVNS does not significantly improve anxiety in alcohol-dependent patients.

Recent animal studies have explored the effects of taVNS on the BDNF levels. Notably, taVNS has been shown to induce neuroprotection against cerebral ischemia/reperfusion injury, which might be related to the upregulation of BDNF expression in the ischemic cortex (45). Consistently, the mean BDNF levels were significantly higher in the patients who received taVNS treatment than in control patients. It is known that BDNF levels are generally decreased in depressive patients and can be increased by antidepressant therapies (46). Correlation analysis showed that BDNF levels were negatively correlated with the 24-item Hamilton Rating Scale for Depression (HAMD-24) scores in elderly patients with refractory depression after repetitive transcranial magnetic stimulation (rTMS), suggesting that rTMS might improve depression by influencing peripheral BDNF levels (47). Similarly, peripheral BDNF concentrations were higher in patients with sleep disorders, and multiple linear regression analysis revealed that peripheral BDNF concentrations were independently correlated with PSQI scores (48). These findings indicate that a close relationship between BDNF, depression (49), and sleep disturbances (50) exists. Taken together, it is rational to deduce that taVNS could alleviate depression symptoms and sleep quality in alcohol-dependent patients after withdrawal, which might be involved in the increased levels of peripheral BDNF.

Plasma and serum BDNF concentrations are both common indicators for evaluating the level of BDNF in peripheral blood (51). Recent studies have consistently reported altered levels of BDNF in the circulation (i.e., serum or plasma) of patients with major depression, bipolar disorder, Alzheimer's disease, Huntington's disease, and Parkinson's disease (52), suggesting that BDNF levels in serum and plasma can be used to reflect the level of BDNF in peripheral blood. Moreover, it has been suggested that plasma BDNF levels are significantly positively correlated with cerebral spinal fluid BDNF levels, which indicates that plasma BDNF levels might represent the BDNF expression profile of the central nervous system (53, 54). Furthermore, a meta-analysis of 52 studies indicates that peripheral BDNF level, better documented in plasma than in serum, is a potential biomarker of disease activity in bipolar disorder (55). Therefore, plasma BDNF levels were selected in the present study.

Several lines of evidence suggest an anti-inflammatory effect of taVNS. First, animal studies have suggested that taVNS could suppress proinflammatory cytokine levels and NF-κB p65 expression in the lung tissues in endotoxemia-affected anesthetized rats (56). Second, clinical evidence has indicated that VNS is associated with marked peripheral increases in pro- and anti-inflammatory circulating cytokines, such as IL-6, TNF-α, and TGF-β in patients with resistant depression (57). However, the downregulation of inflammatory factor levels by taVNS in alcohol-dependent patients was not observed in the present study. The specific mechanism of this discrepancy is not clear, which may be partly due to the different types of diseases.

Although preclinical studies have indicated decreased serum leptin concentrations in normally fed rats (58) and high-fat diet-fed rats (25) that received VNS, there are no reports on clinical studies on the effect of VNS on peripheral leptin levels. Considering that there is a close relationship between blood leptin levels and alcohol craving, we first investigated the plasma leptin concentrations in alcohol-dependent patients after taVNS treatment. Similar to the effect on alcohol craving, taVNS did not change the levels of leptin, indicating that taVNS may not be suitable as a treatment for craving after withdrawal in alcohol-dependent patients.

There are some limitations to this study. First, the current study is a single-center study with a small sample size. Second, the scale scores of the patients were collected only before and after the 4-week treatment. It is more appropriate to collect the scale score information once a week. Third, since benzodiazepine substitution may affect the levels of BDNF (59, 60), the changes in BDNF levels before and after treatment with benzodiazepine should be observed. Fourth, the long-term effects of taVNS on the plasma levels of BDNF, IL-6, TNF-α, and leptin were not observed.

At the end of this study, we concluded that taVNS could improve depression symptoms and sleep quality in alcohol-dependent patients after withdrawal, which might be related to the upregulation of plasma BDNF levels. Further investigation will help identify the exact mechanism of the potential therapeutic benefits of taVNS on PAWS in alcohol-dependent patients after withdrawal.

## Data Availability Statement

The raw data supporting the conclusions of this article will be made available by the authors, without undue reservation.

## Ethics Statement

The studies involving human participants were reviewed and approved by Hefei Fourth People's Hospital. The patients/participants provided their written informed consent to participate in this study.

## Author Contributions

Y-MW, Y-YX, YZ, and L-YX participated in the experimental design and statistical analyses. YZ, Q-QW, WH, YL, and Y-HS performed the experiments. Y-MW and Y-YX drafted the manuscript. Y-YX edited the manuscript. All authors contributed to the article and approved the submitted version.

## Conflict of Interest

The authors declare that the research was conducted in the absence of any commercial or financial relationships that could be construed as a potential conflict of interest.

## Publisher's Note

All claims expressed in this article are solely those of the authors and do not necessarily represent those of their affiliated organizations, or those of the publisher, the editors and the reviewers. Any product that may be evaluated in this article, or claim that may be made by its manufacturer, is not guaranteed or endorsed by the publisher.
